# Research on grading detection methods for diabetic retinopathy based on deep learning

**DOI:** 10.12669/pjms.41.1.9171

**Published:** 2025-01

**Authors:** Jing Zhang, Juan Chen

**Affiliations:** 1 Jing Zhang, Department of Ophthalmology, Affiliated Hospital of Hangzhou Normal University, Hangzhou, Zhejiang, China; 2 Juan Chen, Department of Ophthalmology, Affiliated Hospital of Hangzhou Normal University, Hangzhou, Zhejiang, China

**Keywords:** Diabetic Retinopathy, Deep Learning, Transformer

## Abstract

**Objective:**

To design a deep learning-based model for early screening of diabetic retinopathy, predict the condition, and provide interpretable justifications.

**Methods:**

The experiment’s model structure is designed based on the Vision Transformer architecture which was initiated in March 2023 and the first version was produced in July 2023 at Affiliated Hospital of Hangzhou Normal University. We use the publicly available EyePACS dataset as input to train the model. Using the trained model, we predict whether a given patient’s fundus images indicate diabetic retinopathy and provide the relevant affected areas as the basis for the judgement.

**Results:**

The model was validated using two subsets of the IDRiD dataset. Our model not only achieved good results in terms of detection accuracy, reaching around 0.88, but also performed comparably to similar models annotated for affected areas in predicting the affected regions.

**Conclusion:**

Utilizing image-level annotations, we implemented a method for detecting diabetic retinopathy through deep learning and provided interpretable justifications to assist clinicians in diagnosis.

## INTRODUCTION

Diabetic Retinopathy (DR) has become one of the leading causes of vision impairment among adults worldwide. It is estimated that by the 2040s, about 600 million people globally will have diabetes, with a third of these individuals expected to develop Diabetic Retinopathy.^1^,^2^ Early screening is a crucial step in mitigating the risks of vision impairment or even blindness due to DR. For instance, developed countries like the UK and Iceland have already implemented nationwide DR screening initiatives, with interventions at early stages.^3^,^4^ However, given the vast global population, significant regional differences, and tremendous disparities in medical infrastructure, relying solely on professional physicians for diagnosis cannot meet the expansive need. Therefore, there’s an urgent requirement for cost-effective solutions^5^ that can effectively address this issue.

In recent years, with the significant advancement of artificial intelligence technologies, especially breakthroughs in the domain of deep learning, utilizing deep learning methods to assist individuals with basic medical knowledge in DR diagnosis is gradually becoming a viable approach.^6^ Nonetheless, deep learning-based DR diagnostics currently face several challenges: 1) Early-stage lesions primarily manifest as a few microaneurysms, which even specialists might overlook. 2) The annotation cost for training deep learning models is high^7^, focusing mainly on final diagnostic results (presence or absence of lesions or overall lesion grading, i.e., image-level annotations) and lacks extensive lesion-specific annotations (i.e., lesion-level annotations).

Existing deep learning techniques often provide results indicating only the presence or absence of lesions or an overall lesion grade, without offering interpretable rationales. To address these challenges, we have explored the Transformer^8^ model architecture, which shows promising potential in the deep learning domain. By modifying parts of its network structure and converting the prediction’s intermediate outputs into human-readable formats, we aim to tackle the aforementioned issues.

## METHODS

This study started at the Affiliated Hospital of Hangzhou Normal University in January 2023 and the first version was produced in July 2023.

### Datasets:

The DR images utilized in this study were sourced from several public repositories, and the obtained aggregate data is ascertainable in scenarios with a waiver of informed consent, primarily comprising the following:

### Ethical Approval:

The study was approved by the ethics review committee at the Affiliated Hospital of Hangzhou Normal University (2023(E2)-HS-009, date February 2, 2023).

### EyePACS Dataset^9^:

Maintained by the Department of Ophthalmology and Vitreoretinal Research at Stanford University School of Medicine. The dataset encompasses 35,126 retinal images with varying resolutions and contrast levels. These images were annotated by seven expert physicians based on the severity of DR and are categorized into five major classes: Normal, Mild, Moderate, Severe, and Proliferative. This dataset was employed as the training set for this research.

### Messidor-2 Dataset^10^:

This dataset contains approximately 1,748 images with resolutions of either 1440x960, 2240x1488, or 2304x1536. The images were sourced from three different French medical institutions and were captured using three distinct types of cameras. Each image underwent a clinical-level assessment of diabetic retinopathy by experienced ophthalmologists. This dataset served as the evaluation set for our research.

### APTOS2019 Dataset^11^:

Collected by Aravind Eye Hospital in India. The images in this dataset are derived from individuals residing in rural areas and were annotated by seasoned physicians. Out of these, images and labels of 3,662 eyes were used for training purposes, while the data for the remaining 1,928 eyes was used for testing.

### IDRiD Dataset^12^:

A public dataset provided jointly by Sankara Nethralaya and the Tata Institute of Fundamental Research in Mumbai. The dataset is segmented into three parts, of which the disease grading segment contains 516 annotated graded images. This section was utilized to validate the grading efficacy of the model

### Model Design:

**Fig.1:**
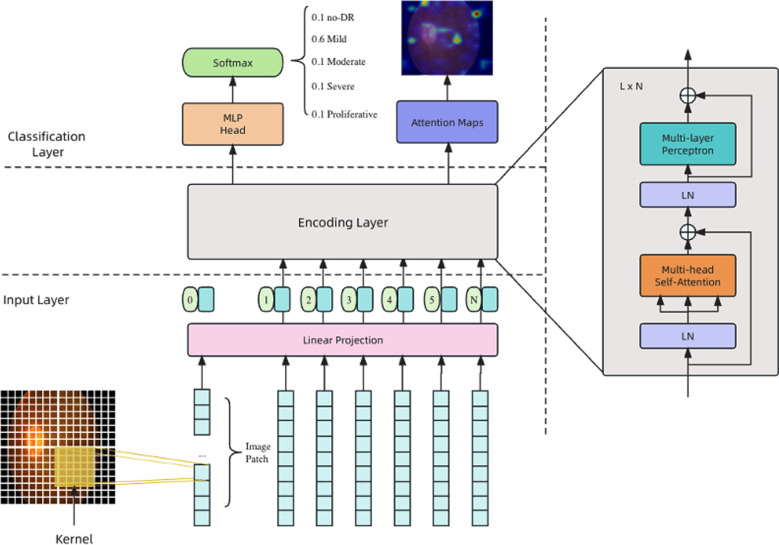
Architecture of the proposed model, highlighting the integration of convolutional local features and global attention mechanisms for diabetic retinopathy detection.

The model architecture of this study was designed based on the Vision Transformer (ViT)^13^ model. It primarily consists of three components: the Input Layer, the Encoding Layer, and the MLP (Multilayer Perceptron) Layer, as illustrated in Fig.1.

### Input Layer:

The original ViT processes image inputs by segmenting them into patches of size 16x16 or 32x32 and linearly embedding them into a higher-dimensional space. However, this approach overlooks the intrinsic local structures within the images, which have been a pivotal factor for the success of many other image processing models, particularly Convolutional Neural Networks (CNNs). To address this limitation, this paper integrates the local structure characteristics of CNNs^14^, replacing the image segmentation process with convolution operations. Specifically, a convolutional kernel of size 16x16 is applied to the image, with the resultant feature maps serving as the model’s input. Each feature map is treated as a token. In doing so, while preserving the global attention mechanism of the ViT, the model benefits from the local receptive field offered by convolutional operations. The process can be represented as follows:

*Patch = MaxPool(ReLu(Conv2d))*

### Encoding Layer:

As depicted on the right side of Fig.1, the encoding layer consists of L encoding blocks. Each encoding block is structured with a Multi-head Self-attention (MSA) unit followed by a Multi-Layer Perceptron (MLP) unit. A Layer Normalization (LN) is incorporated before the inputs of these two units, and a residual connection is added after their outputs, as illustrated on the right of Fig.1. The mathematical representation is as follows:


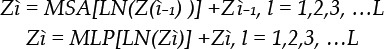


The objective of this paper was not only to detect the disease grade but also to provide the corresponding diagnostic basis. This can be computed from the interaction information between tokens stored in the MSA block. The MSA block is composed of multiple attention heads (assumed to be K in this paper), represented as Self-Attention (SA), with the computation as follows:


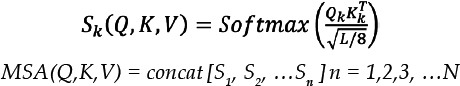


S_k_ represents the attention information of the k-th head; *Q*, *K*, and *V* are computed from the input vector and the weight matrix obtained through training, with the formula as follows:


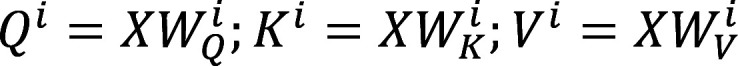


*X* represents input vector, 
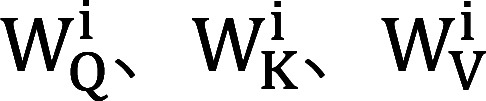
 is the weight matrix learned through training.

### Classification Layer:

As shown in the upper part of Fig.1, the classification layer consists of an MLP block and a Softmax block, as described below.:


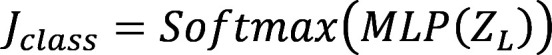


### Training:

### Preprocess:

**Fig.2:**
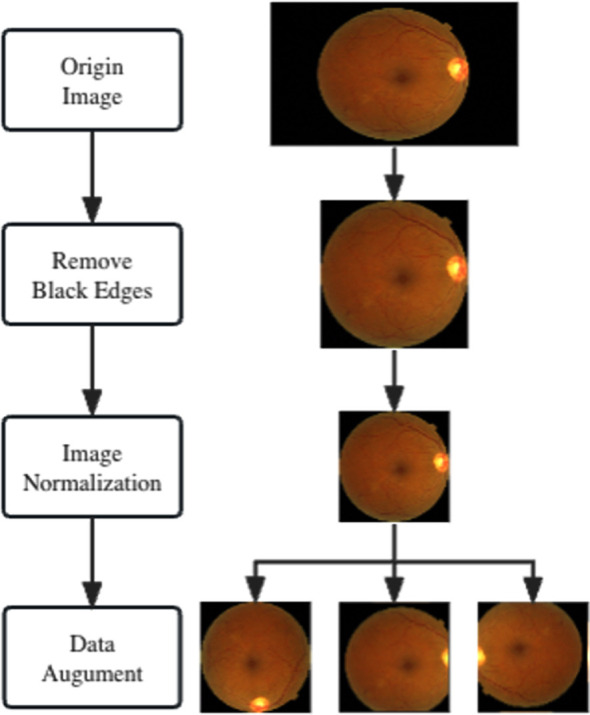
Flow of the preprocessing steps.

Due to the data collection process being conducted by different devices, personnel, and under varying lighting conditions, the images exhibit significant discrepancies in color, brightness, contrast, and resolution. Furthermore, to address the aforementioned issue of uneven sample distribution, we designed the following preprocessing workflow^15^ to enhance the quality of the training data, as depicted in Fig.2.

### Image Normalization:

The original images vary in resolution, brightness, and contrast. Thus, image normalization is employed to confine the pixel values of retinal images within a certain range. In this study, the black edges of the images are first removed, followed by resizing all images to 256X256 dimensions.^16^ There are two reasons for choosing this size: First, pathological features in the images, such as microaneurysms, are very subtle. If the size is too small, these features might be lost, hampering the feature learning of these areas. Second, considering the batch processing capability of the GPU, an overly large size would increase the training difficulty and is not conducive to the training of the network.

### Data Augmentation:

In the dataset used for this study, the distribution of image counts across categories is extremely imbalanced. This research employs data augmentation techniques to balance the dataset distribution. Specifically, images are subjected to random rotations (rotation angles of 90/180/270 degrees)^17^, random translations (translation range of [-15,15]), and random adjustments in brightness and contrast.

### Environment:

The training and validation code for this experiment is written using the PyTorch deep learning framework (version: 1.12.1). The training task was executed on a machine with the following configuration:

- CPU: Intel Xeon (Cascade Lake) Platinum 8269CY.
- RAM: 96GB.
- GPU: Nvidia A100 80GB.

## RESULTS

The objectives of this model consist of two parts: detecting the grade and providing the basis for detection. For these two objectives, different datasets were chosen for validation in this study.

### Grading Result:

For the validation of lesion grade detection results, this study primarily utilized the Messidor-2, APTOS2019, and IDRiD datasets for verification. This study employs widely-used metrics in image recognition for evaluation, namely: Accuracy, Precision, Recall, Specificity, and F1 Score. The results are presented in Table-I.

**Table-I:** Performance of the model on various datasets.

Dataset	Accuracy	Precision	Recall	Specificity	F1 Score
EyePACS	88.01	90.12	89.49	91.32	89.23
Messidor-2	87.35	88.26	89.34	90.72	90.10
APTOS2019	89.35	89.97	91.71	92.58	89.93
IDRiD(Grade)	85.34	87.34	88.26	89.35	86.43

### Lesion Area:

This study conducted validation using the lesion segmentation portion of the IDRiD dataset, with sampling results presented in Table-II.

**Table-II:**
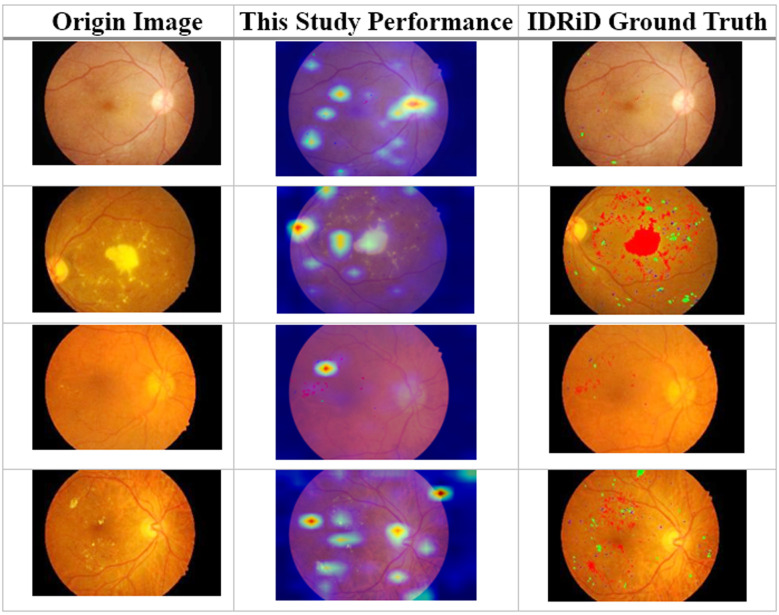
Lesion Area Results.

## DISCUSSION

This study focused on two main objectives: detecting the grade of lesions and providing a basis for this detection. To validate the model’s performance, different datasets were employed, including Messidor-2, APTOS2019, and IDRiD. The model demonstrated robust performance across these datasets, with consistently high scores in key image recognition metrics such as Accuracy (89.35%), Precision (90.12%). This indicates that the model possesses significant potential in the field of medical image analysis, particularly in the accurate grading and identification of lesions, a capability that is currently rare in models that can achieve both of these objectives simultaneously.

Compared with other studies, such as Gulshan et al.^18^ who used CNN-based approaches achieving 93.8% accuracy, our model adds value by not only classifying the presence of lesions but also providing interpretable justifications. This interpretability aspect is less explored in similar models, making our approach particularly valuable in a clinical context where understanding the decision-making process is crucial. Furthermore, studies like Qomariah et al^19^. and Xia et al^20^. have explored segmentation and classification techniques with commendable accuracy, yet they often lack a cohesive strategy that combines lesion grading with transparent decision rationales. Our model addresses these limitations by employing attention mechanisms within the Vision Transformer framework, enhancing the model’s ability to highlight specific areas of concern, thus directly supporting the diagnostic process.

Our study contributes new insights to the medical literature by demonstrating the feasibility of integrating deep learning interpretability in clinical workflows. This approach has significant implications for reducing the burden on healthcare providers, enhancing early detection, and ultimately improving patient outcomes. By providing both diagnostic grading and a visual basis for these grades, our model supports a more holistic and reliable approach to DR screening. The clinical impact of our findings is substantial, offering a pathway to more reliable AI-assisted diagnostics that not only match but potentially exceed the capabilities of traditional screening methods. The enhanced interpretability also paves the way for broader clinical adoption, as it aligns more closely with the needs of medical professionals who require actionable insights rather than opaque predictions.

### Limitations:

Due to the variability in experimental equipment and the technical expertise of operators, there is a significant disparity in the quality of image data collected. To generalize from publicly available datasets to actual operational scenarios, further enhancements are required.

## CONCLUSION

In summary, our model represents a significant advancement in AI-driven diabetic retinopathy screening, merging high accuracy with practical clinical utility. Future work will focus on further validating the model in diverse clinical settings to establish its robustness and explore additional modifications that could further enhance its performance.
